# Introduction: Indology and Aryanism: Knowledge of India in Nazi Germany—An Invitation for New Research

**DOI:** 10.1007/s00048-023-00367-w

**Published:** 2023-09-11

**Authors:** Moritz Epple, Maria Framke, Eli Franco, Horst Junginger, Baijayanti Roy

**Affiliations:** 1 Historisches Seminar, Goethe-Universität Frankfurt, Norbert-Wollheim-Platz 1, 60629 Frankfurt am Main, Germany; 2 Historisches Seminar, Universität Erfurt, Nordhäuser Str. 63, 99089 Erfurt, Germany; 3 Institut für Indologie und Zentralasienwissenschaften, Universität Leipzig, Schillerstraße 6, 04109 Leipzig, Germany; 4 Religionswissenschaftliches Institut, Universität Leipzig, Schillerstraße 6, 04109 Leipzig, Germany

**Keywords:** Indology, Nazi Germany, Deep Orientalism, Aryan Ideology, Indian anti-colonialism, Ludwig Alsdorf, Devendra Nath Bannerjea, Erich Frauwallner, Menaka Ballet, Indologie, Nationalsozialismus, Deep Orientalism, „Ariertum“ als Ideologie, Antikolonialismus in Indien, Ludwig Alsdorf, Devendra Nath Bannerjea, Erich Frauwallner, Menaka Ballett

## Abstract

The introduction to our special issue offers a brief survey of the historical literature on knowledge about India in Nazi Germany and distinguishes three different, but interrelated layers of such knowledge: disciplinary knowledge of Indology as an academic field, knowledge fulfilling the needs of state agencies, and popular knowledge (and beliefs) about India.

## Abstract (de)

Die Einleitung zu unserem Themenheft bietet einen kurzen Überblick über bisherige historische Literatur zum Wissen über Indien im nationalsozialistischen Deutschland und skizziert drei verschiedene, aber miteinander verbundene Schichten solchen Kenntnissen: Disziplinäre Kenntnisse der Indologie als akademisches Fachgebiet, Wissen, das den Bedürfnissen staatlicher Stellen gerecht wird, und Allgemeinwissen (und Überzeugungen) über Indien.

## Deep Orientalism

In his ground-breaking and provocative article “Deep Orientalism? Notes on Sanskrit and Power Beyond the Raj”, Sheldon Pollock reminded us that the notions of “Aryan” and “non-Aryan” descent entered the legal codes and practices of discrimination against Jews and other minorities in Germany only months after the National Socialist Party came to power in early 1933, through the absurdly named *Gesetz zur Wiederherstellung des Berufsbeamtentums *(Law for the Restoration of the Professional Civil Service) of 7 April 1933 and its various regulatory additions and subsequent specifications. In the twelve years that followed, discourses on “Aryan” or “non-Aryan” descent and culture pervaded political circles, legal and administrative practices, and everyday life in Germany just as it shaped a significant segment of German academia. Rarely has a notion originally coined by philologists had such an infamous career. Despite this, our knowledge about the role and involvement of studies of India—through which the notion of an Aryan culture first entered German academic discourse—in Nazi Germany remains surprisingly limited, even three decades after Pollock’s intervention.

The field, whose origins lie both in philology and in broader early nineteenth-century cultural discourses,^1^ remained heterogeneous, comprising both the study of (primarily) ancient Indian texts (a field that eventually came to be called Indology) and, more generally, the academic study of Indian religions, culture and society. Only a small number of scholarly studies have addressed the history of the academic study of India in Nazi Germany and its political contexts since Pollock’s seminal 1993 paper. In it, Pollock raised two interrelated issues by asking us to think both “about German Indology during the years 1933–45 and about forms of precolonial domination in South Asia”. His challenge thus was to relate the “‘orientalist’ ideologemes […] about an Aryan culture of the past” that became so attractive to German authorities after 1933 with a critical analysis of the legitimation of superiority and power found in, or ascribed to, Sanskrit tradition (Pollock 1993: 89). As he pointed out, influential Indologists and scholars of Indian religion and languages, including Walter Wüst in Munich, Ludwig Alsdorf in Münster, Jakob Wilhelm Hauer in Tübingen, Hermann Lommel in Frankfurt, Erich Frauwallner in Vienna, were eager to not only emphasize the affinity of their academic expertise with the claims to Aryan supremacy that Germany’s new government put a premium on, but also to offer their professional services to the state if and when desired. As a characteristic example of these affinities, Pollock pointed to Erich Frauwallner’s contribution “Die Bedeutung der indischen Philosophie” to a conference in Berlin, organized in 1942 within the initiative “Kriegseinsatz der deutschen Geisteswissenschaften” (Wartime deployment of the German humanities) known as *Aktion Ritterbusch*^2^:

“In his presentation, Frauwallner argued that the special meaning of Indian philosophy lay in its being ‘a typical creation of an Aryan people’, that its similarities with western philosophy derived from ‘the same racially determined talent’, and that it was a principal scholarly task of Indology to demonstrate this fact. Reiterating an axiom of NS doctrine, that ‘Wissenschaft in the strict sense of the word is something that could be created only by Nordic Indo-Germans’, Frauwallner adds, ‘From the agreement in scientific character of Indian and European philosophy, we can draw the further conclusion that philosophy as an attempt to explain the world according to scientific method is likewise a typical creation of the Aryan mind’” (Pollock 1993: 93).^3^

While stereotypes such as those invoked by Frauwallner^4^ certainly deserve to be rejected, they should not be forgotten. The involvement of Indology in “Aryan” politics and culture in the Third Reich, and the historical linkages between old and new ideas about Aryan supremacy thus deserve further study. And if Pollock noted with some surprise in 1993 that “no German or indeed, any other Indologist has undertaken an analysis of the field and the relationship of the questions of scholarship and the questions of state since the war” (Pollock 1993: 95), we must admit that, three decades later, we are still far from a comprehensive historical understanding of the relationship between various forms and functions of knowledge (and, more broadly, of cultural beliefs) about India, and the various actors involved, and National Socialism.

## The History of a Field Entangled with Ideology

In surveying the state of the research on these topics, a clarification is important: The term *Indologie* has traditionally been used in German-speaking academic spaces as a broad category encompassing a number of academic fields concerned primarily (but not exclusively) with the study of literary and scholarly texts from ancient India. The problem of defining the academic field of Indology continued to be relevant in the period under consideration here. Although, beginning in the early nineteenth century, some German speaking universities had established professorships exclusively dedicated to Sanskrit studies, in the early twentieth century, Sanskrit, alongside different kinds of ancient Indian literatures (religious, philosophical, historical, scientific, cultural), was also studied as part of the syllabi in various other disciplines, including comparative linguistics (*Vergleichende Sprachwissenschaft*), ancient history and archaeology (*Altertumskunde*), religious studies (*Religionswissenschaft*) and fields that assumed particular importance during the Nazi era, such as *Rassenkunde* and *Völkerkunde, *including the study of “Indo-German” cultural heritage. Moreover, during the 1930s and 1940s, with the rise of the anti-colonial struggle in India and the onset of the Second World War, contemporary Indian affairs gained in importance for the Nazi government and its increasingly anti-British foreign policy. A number of German academics with knowledge of Indian languages and contemporary Indian developments responded by offering their expertise.

Several studies have looked at the participation of some of these disciplines in validating various *völkisch* (“blood and soil”) notions that were congruous with the racial politics of the Nazi regime.

The discourse on Aryanism and Indo-German antiquity was a trope common to both Indology and these academic fields. Still, even in the historiography on the humanities in Nazi Germany that has emerged in recent decades, the issue is rarely considered. Characteristic examples include Frank-Rutger Hausmann’s important studies of the *Aktion Ritterbusch* (Hausmann 2007) and his collection *Die Geisteswissenschaften im ‘Dritten Reich’ *(Hausmann 2011).^5^ As was sometimes pointed out in reviews of this and similar collections, it comes as no surprise that *kleine Fächer *or small disciplines^6^ cannot easily be subjected to same level of historical scrutiny. Nevertheless, given the ideological relevance of the area in question for Nazi politics, the small number of scholarly studies looking at knowledge of India in Nazi Germany is surprising.

Turning to work that has actually been done for the field of Indology, several studies—based mostly on empirical methods, and often in specific local contexts—have focused on three Indologists prominently linked to the Nazi political establishment, all of whom were mentioned in Pollock’s article: Walther Wüst, Jakob Wilhelm Hauer and Erich Frauwallner. Michael Kater discussed Wüst’s role as the curator of Heinrich Himmler’s *Ahnenerbe *as a part of his study on the *Ahnenerbe* (Kater 1966 and later editions).^7^ Maximilian Schreiber (2008) reviewed Wüst’s tenure as professor of Indo-Aryan Studies and Rector of the University of Munich, while Horst Junginger (2008) analyzed his career and writings glorifying Hitler and the Nazi *Weltanschauung*.

The multifaceted career of Jakob Wilhelm Hauer who, apart from being a leader of the German Faith movement, was also a member of the SS, professor of religious studies and Indology at the University of Tübingen and director of Tübingen’s *Arisches Seminar* (an institute whose main duty was to oversee the compatibility of school text books with an “Aryan” world view) has also been critically examined by Hufnagel (2003), Junginger (2003) and (2008) and others.^8^ Similarly, Tübingen remains the best studied place for a history of Indology *including* the years 1933 to 1945 (see Brückner et al. 2003, in which both Hufnagel’s and Junginger’s 2003 chapters were published).

As mentioned above, Nazi politics also impacted the career of Indologist Erich Frauwallner, who joined the NSDAP in Vienna in 1932, before it was prohibited in Austria in 1933 until the *Anschluss* in 1938. In the year of the *Anschluss*, Frauwallner became an associate professor at the University of Vienna, replacing Bernhard Geiger, a scholar of Iranian studies who also specialised in Indology and was dismissed due to his Jewish descent. In 1942, Frauwallner was promoted to the directorship of Vienna’s institute of Oriental Studies. The connections between his worldview, political affiliation and professional work were the subject of a detailed study by Jakob Stuchlik (2009).

Frank Neubert (2012) has studied a curious controversy between the Leipzig Indologist Johannes Hertel and Mathilde Ludendorff that erupted in 1932 when Hertel attacked Ludendorff for her claims that Christianity was based on the plagiarism of ancient Indian ideas. The controversy played out before the German courts until the mid-1930s; Neubert uses the proceedings to document how both sides mobilized motives of the ideological discourses of the period.

In an edited volume on Indian studies at various institutions in Berlin (Framke et al. 2014), Maria Framke contributed an article on the relationship between the careers of various academics and the Nazi regime. Her brief discussion focussed on the Indologists Bernhard Breloer and Ludwig Alsdorf, as well as the Indian lecturer Devendra Nath Bannerjea, who taught a number of courses related to contemporary India at the Seminar for Oriental languages and, from 1935 onwards, at the *Staatswissenschaftliche Fakultät* of Berlin’s Friedrich Wilhelms Universität.

On a less local level, Baijayanti Roy’s handbook entry (2017) surveyed the porous relationship between aspects of Indological scholarship and *völkisch *ideas in Germany before and during the Nazi period. Another helpful summary of existing research was recently provided by Douglas McGetchin (2021).

## Forms and Functions of Knowledge About India: The Four Essays

The following pages encompass four essays that take up Pollock’s challenge and address different forms and functions of knowledge about India in Nazi Germany. The essays look at the activities and careers of prominent Indologists and “India experts” in National Socialism, at the activities of institutions in Germany concerning India, at German war interests in India under British rule, and at the role German Indologists played in pursuing these interests. The essays also look at the image of India, both popular and academic, that was cultivated in Germany during the Nazi period. Finally, they discuss the difficult memory of the academic discipline of Indology in post-war Germany. All contributions were first presented and discussed at a workshop at the Goethe University in Frankfurt am Main on 31 January 2020, “Knowledge of India in Nazi Germany”, organized by the research project *Indology in National Socialist Germany, *funded by the Deutsche Forschungsgemeinschaft and set up by the Working Group in the History of Modern Science at Goethe University in Frankfurt am Main, in cooperation with Eli Franco at Leipzig University.^9^

Each of the papers also addresses one of the various layers of knowledge about India in Nazi Germany. The first layer is the *disciplinary knowledge *of Indology as an academic field, the knowledge generated and taught by members of the academic profession as part of what was then considered to be Indology. With respect to this body of knowledge, the intersection of some of its constituent elements with the ideological discourses circulating during National Socialism as well as the forms in which its academic producers interacted and exchanged resources with the state and its agencies must be analyzed.^10^ A second type of knowledge needs historical scrutiny: The knowledge directly involved in the exchange of resources between academic experts and the political arena. It was provided by actors trained in the academic discipline of Indology who used their skills and expertise not to contribute to the discipline’s body of knowledge, but to fulfil the *knowledge needs of state agencies* such as the foreign ministry or branches of the military.^11^ Here, linguistic skills pertaining to spoken Indian languages often stood at the centre, along with a wider cultural and political knowledge of contemporary India and its internal dynamics during a period in which the British Empire was gradually losing its colonizing influence. As the contributions to this special issue attest, both German and Indian actors contributed to this second type expertise. Finally, the third layer that comes to the fore is *popular knowledge*, or rather the beliefs, images and imaginations about Indian culture that circulated among political elites, the media and segments of the general population in Nazi Germany. These different layers often interacted, but were not without mutual tensions: As we will see (and as we know from rather similar cases in the present), academic expertise and popular beliefs about Indian culture at times disagreed or stood outside the immediate knowledge needs of government agencies.

Eli Franco’s essay begins by looking at the first layer, and by directly addressing the problems underlying the conflicts internal to the discipline of Indology in dealing with its past. Franco highlights Jakob Stuchlik’s exemplary efforts in *Der arische Ansatz. Erich Frauwallner und der Nationalsozialismus* (2009) to uncover, through consummate archival work, new details about Frauwallner’s involvement with Nazi institutions such as the NSDAP, the SA and the Gestapo, and his (unsuccessful) attempt to appropriate confiscated Jewish real estate for himself. Franco then supplements Stuchlik’s analysis of Frauwallner’s racialized or racist theories on Indian philosophy and Buddhism. In the second part, his contribution considers Walter Slaje’s scandalous review of Stuchlik’s research, which tried to whitewash Frauwallner and his work despite his adoption of Nazi ideology, antisemitism and deep involvement with Nazi institutions. The role of academic knowledge in Nazi Germany is at stake here, in connection with the intricacies of a biography deeply immersed in and supportive of the “Third Reich”.

The articles by Baijayanti Roy and Maria Framke, in turn, focus on attempts to fulfil the knowledge needs of state agencies. Roy does so by tracing a phase in the career of Ludwig Alsdorf (1904–1978). Whereas today, Alsdorf is mostly remembered as a pioneering scholar of narrative texts pertaining to the ancient monastic religion of Jainism, the essay discusses Alsdorf’s role as an expert of modern India in the Third Reich. Between 1930 and 1932, Alsdorf taught at Allahabad University in India. Upon his return to Germany in 1932, Alsdorf joined the NSDAP and some of its subsidiaries after 1933. Political contacts acquired through these engagements as well as his claims to first-hand experience of India secured Alsdorf an assignment to write a book on modern India for the NSDAP’s external affairs department. The success of this book, which aimed to provide information about India’s colonialization to state authorities for the purpose of propaganda, established Alsdorf’s reputation as an expert on modern India and procured him employment at the foreign ministry. Alsdorf also mediated between information collected by secret agents on developments in India and representatives of the German state who used this knowledge as part of their larger strategies. In the process, he became a gate keeper, directly influencing what was allowed to be published in Germany on contemporary India.

Maria Framke’s contribution addresses closely related issues through the lens of Devendra Nath Bannerjea, an Indian anti-colonial intellectual and adventurer trying to find his way in Germany during the Nazi period. Predicting the breakup of the British Empire and the emergence of an independent India, representatives of the German government felt that Germany needed specialised and detailed “practical” knowledge of India to ensure fruitful political, economic and intellectual relations in future. Although Ludwig Alsdorf was considered the ideal candidate for the position, he had a self-appointed rival to this office: Devendra Nath Bannerjea. Using his life and work as a starting point, this article considers the larger question of knowledge production for the National Socialist state on two levels: One, it examines what kind of knowledge was perceived by whom as useful, and which specific contributions were made by Bannerjea. Two, it focuses on the changing nature of Bannerjea’s political and personal concerns, especially in comparison to the tasks assigned to him, his competition with Alsdorf, and the organizing principles of the NS regime.

The fourth contribution, by Isabella Schwaderer, takes up the rarely considered layer of popular knowledge about India by analysing public reactions and debates around a series of performances of the Indian ballet *Menaka,* which toured Germany and neighbouring countries from 1936 until early 1938. The contemporary reviews of these performances were quite diverse, ranging from imaginative exotic speculations to highly emotional descriptions of transcendental aesthetic experiences and scholarly ethnographic descriptions. An analysis of these reviews reveals the diverse popular and scientific discourses on topics concerning India circulating at the time, whereby racial theory was taken up just as naturally as ideas of *völkische Kunst*. The performances of this Indian ballet evoked a wide range of different associations that referred to a long tradition of racialized perceptions of Indian culture. The article thus underlines that the production of knowledge about India was given a special role in the National Socialist context, but that the ways in which this role was shaped by individual actors varied significantly.

## Open Questions and Further Issues

To conclude this introduction, we would like to briefly raise a number of questions that the present essays confront only cursorily, but which should be addressed in future research on the subject. One important task, only briefly and incompletely explored in Pollock’s study,^12^ is to assess the changes in academic personnel after the Nazi rise to power in 1933. Who among those working in Indology were dismissed, robbed of career opportunities or their lives in Germany, or the countries it occupied? How were their colleagues, particularly from the same academic field, involved in these changes, how did they react, or even profit from them?

Second, and in connection with this task, we need to ask more broadly, how did Indologists and experts on India—and, in particular, those who remained in Germany—choose their academic, institutional and political affiliations during the Third Reich? This question covers a wide range of subtopics, and leads back to the essential but complex issue of the exchange of resources between academic actors and the state—be they intellectual, ranging from language skills to legitimizing the state’s engagement with India’s anti-British movements, or material, from the acquisition of funds and office space to the printing of propaganda materials. The above studies address *some* of these questions for a very limited number of actors; much more research is required. In particular, any comprehensive understanding of this aspect must analyse the *networks* involved in the exchanges of resources in which Indologists and scholars of Indian studies participated. It should be clear that a purely disciplinary approach is insufficient—these networks, which connected both to government activities such as the *Aktion Ritterbuch* or the foreign ministry at large, and to academic domains in which experts on India were integrated (see above), extended beyond the discipline. It is thus vital to assess the roles of actors such as Hauer, Alsdorf, Frauwallner or Bannerjea in these larger networks.

A third and crucial question centres around the role of the “ideologemes” produced by Indologists, as Pollock aptly put it. Whereas engineers, mathematicians, physicists or chemists could offer their expertise to the state with a certain degree of ideological independence, at least in some cases,^13^ this dynamic needs closer inspection for the field of Indology. While it might be the case that some academics offered their expertise to the state for various pragmatic reasons, there is simply no way around the fact that the discourse surrounding the Aryan/non-Aryan distinction formed one of the essential, and most deadly, strands of Nazi ideology—whatever distance academics ultimately claimed in retrospect. In terms of the production of Aryan ideologemes, a well-known issue from studies of science and technology in Nazi Germany reappears, namely the issue of self-mobilization. Were the knowledge actors in Indology and studies of India driven by forces beyond their control, or did they themselves constitute a driving force, in the Gramscian sense of an organic intellectual? Was the individual scholar a ‘subaltern’ or a contributor to ideological hegemony?

The historical scrutiny of a fourth dimension may turn out to be the most challenging, namely an analysis of how the transformation of all of the above, from persecution and shifts in academic personnel to political interactions and ideological commitments, changed the dynamics of disciplinary knowledge. Which topics and themes were lost? Which new topics and themes appeared? How was the body of established knowledge affected? In what ways did it change at all?

While the contributions to this small collection can by no means claim to cover the discussion of all aspects of the history of Indology and Indian studies during the Nazi period—indeed, the discussion has barely begun—they aim to reopen the field for a new round of historical discussion and scholarly research.

## Footnotes

1 In his 1993 paper, Pollock proposed to term the latter strand in the emerging field of German Indology “romanticism-Wissenschaft”, a notion that has sparked much debate and is connected to similar contemporary developments in other areas of the humanities and the sciences. Pollock contrasted it with (mainly British) colonialist and Christian missionary interests in gathering knowledge about India and about Indian languages. Since our main interest lies in German developments in the period from 1933 to 1945, we refer interested readers to other studies of nineteenth century Indology and its related fields, both in Germany and elsewhere, including the monographs by Indra Sengupta (2005), Suzanne Marchand (2009), Douglas T. McGetchin (2009), and Markus Messling (2016). Also useful for the early period is the classic study by Ernst Windisch (1917–1920). The history of the myth of “Aryan origins” *before* the Nazi era has led to a second relevant body of literature, from Leon Poliakov’s *The Aryan Myth *(1971) to Sieferle (1987), Römer (1985), von See (1994), Trautmann (1997), Figueira (2002), and Roy (2018).

2 For an analysis of the *Aktion Ritterbusch*, see Hausmann (2007).

3 Pollock quoting Frauwallner’s “Die Bedeutung der indischen Philosophie” in Hans Heinrich Schaeder, ed. 1944. *Der Orient in Deutscher Forschung. *Vorträge der Berliner Orientalistentagung, Herbst 1942. Leipzig: Harrassowitz, 158–169.

4 These racialized readings of the supposed “Aryan” inclination towards philosophy and science were not new. Frauwallner was aware of this and endorsed similar claims made by others, including Wolfram von Soden.

5 Other notable contributions to these fields are Römer (1985), Hutton (1999), Maas (2016) on linguistics; Schulze et al. (1999), Haar (2000) on history; Losemann (1977) and Chapoutot (2014) on classical studies, Junginger (1999 and 2007a), Heinrich (2002) on religious studies. Important collections include Elvert & Nielsen-Sikora (2009) on *Kulturwissenschaften, *and Bialas & Rabinbach (2007) on philosophy, history, and a variety of other disciplines in the humanities. With the exception of Junginger’s publications, few if any of these address the issues discussed here.

6 Here and in the following we adhere to this official English translation of *Kleine Fächer *offered by the German Federal Ministry of Education and Research (BMBF).

7 Kater’s monograph, written under significant pressure from former protagonists and well before the history of the sciences and the humanities of Nazi Germany received closer attention and greater theoretical reflection, must be regarded as dated in several respects; the *SS-Ahnenerbe* is in sore need of a new and more sophisticated historical study. On Kater, see Junginger (2007b: 107).

8 Other studies of Hauer are Nanko (1993), Baumann (2005) and Kubota (2005).

9 The main researcher on this project is Dr. Baijayanti Roy. The findings of her research are scheduled to be published in a monograph; as with most academic projects, the recent pandemic has delayed archival research for this project.

10 Here we obviously make use of Mitchell Ash’s important categories for analysing the mutual interactions between academic fields and political systems (2002).

11 On the history of the foreign office, see Conze et al. (2010); on the so-called *Auslandswissenschaften*, see Botsch (2005). Baijayanti Roy’s forthcoming monograph covers several instances where academic knowledge on India was supplied to state agencies, including the so-called Indian legion.

12 Sheldon Pollock already provided a short list of individual cases of persecuted indologists. It includes “Bette Heimann [emigrated], Walter Neisser [suicide, 1941], Walter Ruben [emigrated], Isidore Scheftelowitz [emigrated], Richard Simon [died 1934], Moritz Spitzer [fate unknown], Otto Stein [died in Lodz Ghetto, 1942], Otto Strauss [died in flight in Holland, 1940], Heinrich Zimmer [emigrated]” (Pollock 1993: 95). Some information can also be found in Stache-Rosen & Stache-Weiske (1990). The case of Heinrich Zimmer, a victim of the Nazis who tried to resist his fate by offering various services to the Nazi regime, is discussed in some detail in Roy (2022).

13 A characteristic example is Ludwig Prandtl, a leading expert in fluid mechanics, who never felt compelled to join the NSDAP but could nonetheless become the president of the all-important *Forschungsführung *installed by the Reich Aviation Ministry in 1942. Even then, a closer analysis often reveals ideological commitments that were not enforced by the state and hence are even more telling as Michael Eckert (2017, Chapters 7 and 8) has shown in Prandtl’s case.
